# Platelet–Lymphocyte Ratio as a Useful Predictor of the Therapeutic Effect of Neoadjuvant Chemotherapy in Breast Cancer

**DOI:** 10.1371/journal.pone.0153459

**Published:** 2016-07-29

**Authors:** Yuka Asano, Shinichiro Kashiwagi, Naoyoshi Onoda, Satoru Noda, Hidemi Kawajiri, Tsutomu Takashima, Masahiko Ohsawa, Seiichi Kitagawa, Kosei Hirakawa

**Affiliations:** 1 Department of Surgical Oncology; Osaka City University Graduate School of Medicine, Osaka, Japan; 2 Department of Diagnostic Pathology; Osaka City University Graduate School of Medicine, Osaka, Japan; 3 Department of Physiology, Osaka City University Graduate School of Medicine, Osaka, Japan; Sudbury Regional Hospital, CANADA

## Abstract

**Background:**

The peripheral blood platelet–lymphocyte ratio (PLR) has been proposed as an indicator for evaluating systemic inflammatory responses in cancer-bearing patients. While some reports suggest a correlation between PLR and prognosis, few studies have examined the relationship between PLR and sensitivity to chemotherapy. We conducted a study on whether PLR could serve as a predictor of the therapeutic effects of neoadjuvant chemotherapy (NAC).

**Methods:**

PLR was evaluated in 177 breast cancer patients treated with the NAC 5-fluorouracil, epirubicin and cyclophosphamide, followed by weekly paclitaxel and subsequent curative surgery. The correlation between PLR and prognosis, and between PLR and the efficacy of NAC, were evaluated retrospectively.

**Results:**

The low PLR group had significantly more patients > 56 years old (p = 0.001) and postmenopausal women (p = 0.001) than the high PLR group. The low PLR group also had a higher pathologic complete response (pCR) rate (p = 0.019). On examining the correlation with prognosis, the low-PLR group was found to have significantly longer disease-free survival (p = 0.004) and overall survival (p = 0.032) than the high PLR group. Multivariate analysis also revealed that lymph node metastasis (*p* = 0.043, hazard ratio = 4.40) and a high PLR (*p* = 0.005, hazard ratio = 2.84) were independent, unfavorable prognostic factors.

**Conclusions:**

For patients with breast cancer treated with NAC, a low PLR indicated high chemotherapy sensitivity, suggesting that PLR could serve as a predictive marker of the therapeutic effect of NAC.

## Introduction

In recent years, the peripheral blood neutrophil–lymphocyte ratio (NLR) and the platelet–lymphocyte ratio (PLR) have been proposed as indicators for evaluating systemic inflammatory responses in cancer-bearing patients and have shown utility as predictive markers for prognosis [1–7]. Neoadjuvant chemotherapy (NAC) increases the rate of breast-conserving surgery and reduces the risk of postoperative recurrence in patients with resectable breast cancer [8–11]. The main purpose of NAC is its use in building a treatment strategy based on confirmed therapeutic effects and their outcomes through reducing tumor size and improving breast-conservation rates [8, 12, 13]. To date, we have reported on the usefulness of NLR as a predictor of the effect of NAC in triple-negative breast cancer [6, 7].

Meanwhile, the PLR in preoperative peripheral blood reportedly represents an independent prognostic factor in breast cancer [6, 7]. A high PLR in many types of cancer, including gastric [14], colorectal [15–17], pancreatic [18] and ovarian [19, 20], is said to indicate a poor prognosis. Platelets are cells containing the largest quantity of growth factors, such as platelet-derived growth factor (PDGF) [21–25], transforming growth factor (TGF)-β [26, 27] and platelet-derived endothelial cell growth factor (PD-ECGF) [28–30]. These platelet-derived growth factors are often produced in large quantities by cancer cells and contribute to cancer growth and histology [25, 26]. Lymphocytes are known to be responsible for the immune response against tumors, suggesting that PLR may be related to prognosis and chemotherapy sensitivity. While some reports suggest a correlation between PLR and prognosis [6, 7, 15, 18, 19], few studies have examined the relationship between PLR and sensitivity to chemotherapy. We conducted a study on whether PLR could serve as a predictor of the therapeutic effects of NAC.

## Materials and Methods

### Patient Background

As described in detail previously [31], a total of 177 patients with resectable, early-stage breast cancer diagnosed as stage IIA (T1, N1, M0 or T2, N0, M0), IIB (T2, N1, M0 or T3, N0, M0) or IIIA (T1-2, N2, M0 or T3, N1-2, M0) were treated with NAC between 2007 and 2013. Our previous report also used the same patient population as the present study, but it was a study of the significance of androgen receptor expression [31]. Tumor stage and T and N factors were stratified based on the TNM Classification of Malignant Tumors, Union for International Cancer Control (UICC) Seventh Edition [32]. Breast cancer was confirmed histologically using core needle biopsies and staged with systemic imaging studies using computed tomography (CT), ultrasonography (US) and bone scintigraphy. Breast cancer was classified into subtypes according to the immunohistochemical expression of the estrogen receptor (ER), progesterone receptor (PR), epidermal growth factor receptor 2 (HER2), and Ki67. Cutoffs for ER and PR positivity were both >0% positive tumor cells with nuclear staining. Tumors with 3+ HER2 on immunohistochemical staining were considered to show HER2 overexpression; tumors with 2+ HER2 were further analyzed using fluorescence *in situ* hybridization; and those with HER2/CEP17 ≥2.0 were also considered to exhibit HER2 overexpression. A Ki67-labeling index ≥14% tumor cells with nuclear staining was determined to be positive.

All patients received a standardized protocol of NAC consisting of four courses of FEC100 (500 mg/m^2^ fluorouracil, 100 mg/m^2^ epirubicin and 500 mg/m^2^ cyclophosphamide) every 3 weeks, followed by 12 courses of 80 mg/m^2^ paclitaxel administered weekly [33, 34]. Forty-five patients had HER2-positive breast cancer and were additionally administered weekly (2 mg/kg) or tri-weekly (6 mg/kg) trastuzumab during paclitaxel treatment [35]. All patients underwent chemotherapy as outpatients.

Therapeutic anti-tumor effects were assessed according to the Response Evaluation Criteria in Solid Tumors (RECIST) criteria [36]. Pathologic complete response (pCR) was defined as the complete disappearance of the invasive components of the lesion with or without intraductal components, including in the lymph nodes. Patients underwent mastectomy or breast-conserving surgery after NAC. All patients who underwent breast-conserving surgery were administered postoperative radiotherapy to the remnant breast. The postoperative adjuvant therapy performed endocrine therapy and/or anti-HER2 treatment depending on every case.

Overall survival (OS) time was defined as the period from the initiation of NAC to the time of death from any cause. Disease-free survival (DFS) was defined as freedom from all local, loco-regional and distant recurrences. All patients were followed up with a physical examination every 3 months, US every 6 months, and CT and bone scintigraphy annually. The median follow-up period for the assessment of OS was 3.4 years (range, 0.6–6.0 years) and for DFS, it was 3.1 years (range, 0.1–6.0 years).

### Ethics Statement

This research conformed to the provisions of the 1995 Declaration of Helsinki. All patients were informed of the investigational nature of this study and provided their written informed consent. The Ethics Committee of Osaka City University approved the study protocol (#926).

### Blood Sample Analysis

Peripheral blood was obtained at the time of diagnosis, before NAC. The number of blood cells was determined using a hemocytometer. Percentages of different cell types were determined using a Coulter LH 750 Hematology Analyzer (Beckman Coulter, Brea, CA, USA). PLR was calculated from the preoperative blood sample by dividing the absolute platelet count by the absolute lymphocyte count. On the basis of previous studies, a PLR value of 150.0 was used as the cutoff value to discriminate between high-PLR (≥150.0) and low-PLR (<150.0) [37].

### Statistical Analysis

Statistical analysis was performed using the SPSS version 19.0 statistical software package (IBM, Armonk, NY, USA). We examined associations between PLR and clinicopathologic variables sing the chi-squared test (or Fisher’s exact test when necessary). The association with survival was analysed using the Kaplan–Meier plot and log-rank test. The Cox proportional hazards model was used to compute univariable and multivariable hazard ratios (HR) for the study parameters with 95% confidence intervals (c.i.), and used in a backward stepwise method for variable selection in multivariate analysis. In all of the tests, a *p*-value of less than 0.05 was considered statistically significant. Cut-off values for different biomarkers included in this study were chosen before statistical analysis.

## Results

In this study, NAC was administered to 177 patients with early-stage breast cancer. The therapeutic effect was pCR in 67 patients (37.9%) and non-pCR in 110 patients (62.1%) (Table 1). PLR was determined in every sample and ranged from 40.5–463.0 (mean, 148.6; median, 135.2; standard deviation, 66.6). The high-PLR group comprised 67 patients (37.9%) and the low-PLR group comprised 110 patients (62.1%). Patients who received NAC were sorted into high- and low-PLR groups and the clinicopathological characteristics of each group were examined. The low-PLR group had significantly more patients > 56 years old (the median value of age) (p = 0.001) and postmenopausal women (p = 0.001) than the high-PLR group. The low-PLR group also had a higher pCR rate (p = 0.019) (Table 2). However, no correlation was seen with other clinicopathological factors, including subtype.

**Table 1:** Clinical response rate and pathological response rate to neoadjuvant chemotherapy.

pathological response	all breast cancers (n = 177)	TNBC (n = 61, 34.5%)	HER2BC (n = 36, 20.3%)	HRBC (n = 80, 45.2%)
pCR: pathological complete response	67 (37.9%)	28 (45.9%)	18 (50.0%)	21 (26.3%)
non-pCR: non-pathological complete response				
PR: partial response	84 (47.5%)	21 (34.4%)	15 (41.7%)	48 (60.0%)
SD: stable disease	19 (10.7%)	8 (13.1%)	3 (8.3%)	8 (10.0%)
PD: progressive disease	7 (3.9%)	4 (6.6%)	0 (0.0%)	3 (3.7%)
RR (CR+PR): response rate	151 (85.3%)	49 (80.3%)	33 (91.7%)	69 (86.3%)

TNBC, triple-negative breast cancer. HER2BC, human epidermal growth factor receptor 2-enriched breast cancer. HRBC, hormone receptor-positive breast cancer. CR, complete response. PR, partial response. SD, stable disease. PD, progressive disease.

**Table 2:** Correlation between clinicopathological features and platelet–lymphocyte ratio in 177 all breast cancers.

Parameters	PLR (n = 177)		p value
	High (n = 67)	Low (n = 110)	
Age at operation			
≤56	44 (65.7%)	43 (39.1%)	
>56	23 (34.3%)	67 (60.9%)	0.001
Menopause			
Negative	38 (56.7%)	34 (30.9%)	
Positive	29 (43.3%)	76 (69.1%)	0.001
Tumor size			
≤2 cm	8 (11.9%)	16 (14.5%)	
>2 cm	59 (88.1%)	94 (85.5%)	0.623
Lymph node status			
Negative	17 (25.4%)	24 (21.8%)	
Positive	50 (74.6%)	86 (78.2%)	0.587
Nuclear grade			
1, 2	53 (79.1%)	84 (76.4%)	
3	14 (20.9%)	26 (23.6%)	0.672
Ki67			
≤14%	32 (47.8%)	42 (38.2%)	
>14%	35 (52.2%)	68 (61.8%)	0.210
Intrinsic subtype			
TNBC	25 (37.3%)	36 (32.7%)	
non-TNBC	42 (62.7%)	74 (67.3%)	0.533
Pathological response			
pCR	18 (26.9%)	49 (44.5%)	
non-pCR	49 (73.1%)	61 (55.5%)	0.019

PLR, platelet–lymphocyte ratio. TNBC, triple-negative breast cancer. pCR, pathologic complete response.

**Fig 1:**
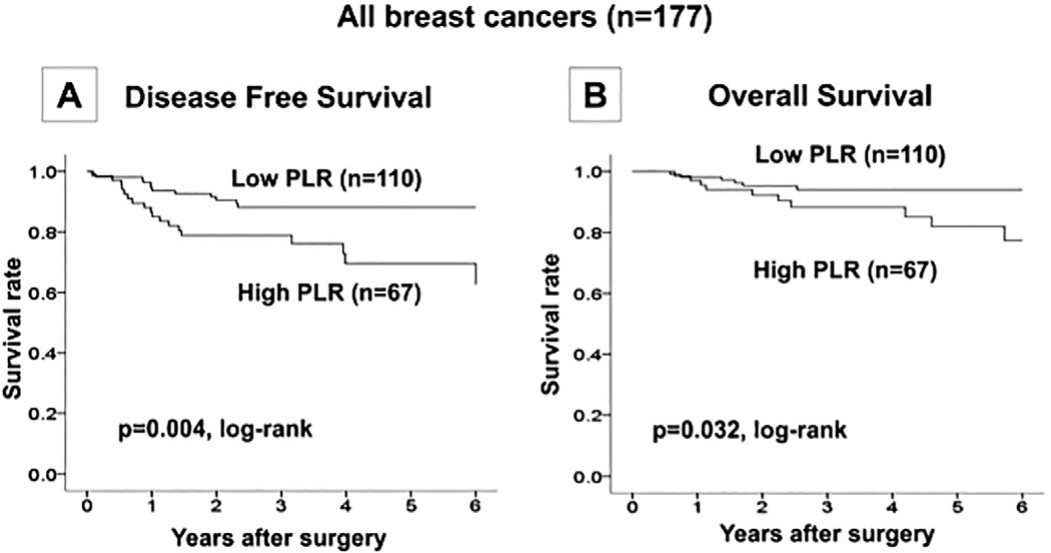
Examination of the correlation with prognosis. The low-PLR group was found to have significantly longer disease-free survival (p = 0.004, log-rank) (A) and overall survival (p = 0.032, log-rank) (B) than the high PLR group.

**Fig 2:**
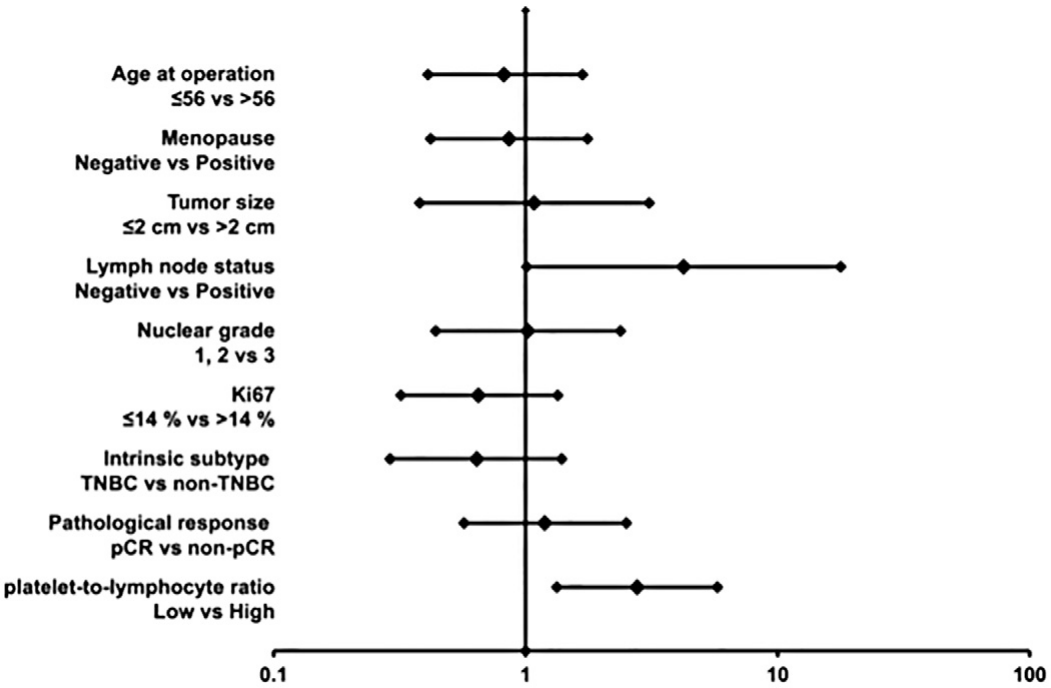
Forest plots. Univariate analysis revealed that absence of lymph node metastasis and a low PLR were good prognostic factors.

On examining the correlation with prognosis, the low-PLR group was found to have significantly longer DFS (p = 0.004, log-rank) (Fig 1A) and OS (p = 0.032, log-rank) (Fig 1B) than the high PLR group. Univariate analysis revealed that lymph node metastasis (*p* = 0.049, hazard ratio = 4.23) and a high PLR (*p* = 0.006, hazard ratio = 2.77) were unfavorable prognostic factors (Fig 2). Moreover, multivariate analysis also revealed that lymph node metastasis (*p* = 0.043, hazard ratio = 4.40) and a high PLR (*p* = 0.005, hazard ratio = 2.84) were independent, unfavorable prognostic factors (Table 3).

**Table 3:** Univariable and multivariable analysis with respect to disease-free survival in breast cancer.

	Univariable analysis			Multivariable analysis		
Parameter	Hazard ratio	95% c.i.	p value	Hazard ratio	95% c.i.	p value
Tumor size (cm)						
≤2 vs >2	1.06	0.37–3.05	0.911			
Menopause						
Negative vs Positive	0.84	0.41–1.73	0.637			
Hormone receptor						
Negative vs Positive	1.05	0.51–2.16	0.886			
HER2						
Negative vs Positive	0.695	0.27–1.82	0.459			
Nuclear grade						
1–2 vs 3	1.03	0.44–2.39	0.954			
Ki67 (%)						
≤14 vs >14	0.65	0.32–1.33	0.238			
Pathological response						
pCR vs non-pCR	0.61	0.28–1.34	0.217	0.75	0.34–1.66	0.475
Lymph node status						
Negative vs Positive	4.23	1.01–17.78	0.049	4.40	1.05–18.48	0.043
PLR						
High vs Low	2.77	1.33–5.76	0.006	2.84	1.37–5.91	0.005

c.i., confidence interval. HER2, human epidermal growth factor receptor 2. pCR, pathological complete response. PLR, platelet–lymphocyte ratio.

## Discussion

In the past it was believed that cancer cells could autonomously proliferate and survive because of a variety of genetic abnormalities, but recently it has become clear that the peripheral environment (tumor microenvironment) greatly affects cancer cells and contributes to the formation of characteristics particular to cancer. Therefore, monitoring the host's tumor microenvironment is believed to play a key role in predicting therapeutic efficacy and prognosis. Systematic inflammatory indicators, such as NLR or PLR, have been reported as monitoring the host's tumor microenvironment. [14,37,38].

The cell proliferation process, particularly autonomous proliferation, is important to elucidate the mechanisms of carcinogenesis, as growth factors and their receptors are closely related to cancer genes [24]. Platelet-derived growth factors include PDGF [21, 22], TGF-β [26, 27] and PD-ECGF [26, 29, 38], and are involved in the repair and regeneration of tissue. PDGF has a similar structure to the protein that makes up the *sis* cancer gene, and the tyrosine kinase structure seen in PDGF receptors is found in some cancer genes. Moreover, administration of PDGF induces cancer genes such as *myc* and *fos*, which are present in the cell nucleus. This suggests that abnormalities that occur in the growth factor activation process are linked to the canceration of cells [25]. TGF-β works to inhibit cancer through cytostatic activity in the early stages of carcinogenesis; however, it conversely works to promote cancer in the later stages as the cancer develops [26].

Platelet-derived growth factors are frequently created in large quantities by cancer cells and have an effect on the histology of cancer [25, 26, 38]. They have been found to contribute to metastasis, invasion and growth of the primary tumor. Peripheral blood platelet count may thus be an indicator of tumor activity. Meanwhile, lymphocytes are responsible for the immune response to tumor growth, and peripheral blood lymphocyte count is thought to be an indicator of tumor suppression. This would suggest that patients in a low-PLR group, with a low platelet count and high lymphocyte count, exhibit high antitumor activity, which would be correlated with a good prognosis and chemotherapy sensitivity. The patients in the low-PLR group in the present study showed a correlation between PLR and the pCR rate, which was an independent, good prognostic factor.

The mechanism whereby PLR and chemotherapy sensitivity are correlated is thought to occur as follows. Platelets are cells that contain the largest quantity of growth factors, and platelet count is an indicator of cancer activity. A low platelet count suggests cancer with low activity. Chemotherapy promotes myelosuppression and lowers the platelet count. Furthermore, chemotherapy increases lymphocyte count by activating the immune response. This is thought to relatively lower the PLR and enhance the antitumor effects.

We have reported on the usefulness of NLR as a predictor of the effect of NAC in triple-negative breast cancer [6, 7]; however, no correlation was seen between PLR and NAC sensitivity with subtype. NLR is useful as an effective predictive marker in a high lymphocyte activity subtype such as triple-negative breast cancer. On the other hand, PLR was not useful as an effect predictive marker according to subtype, because of growth factor participation. The indication criteria of NAC should be decided by progress degree and intrinsic subtype. However, the high-PLR group that an effect cannot expect so much may become an adjuvant biomarker.

## Conclusions

In NAC treatment of breast cancer, a low PLR indicated high chemotherapy sensitivity, suggesting that PLR could serve as a predictive marker of the therapeutic effect of NAC.
